# Structural Potting of Large Aeronautic Honeycomb Panels: End-Effector Design and Test for Automated Manufacturing

**DOI:** 10.3390/ma15196679

**Published:** 2022-09-26

**Authors:** Carlos Campos, Enrique Casarejos, Abraham Segade

**Affiliations:** 1 CINTECX, Department of Mechanical Engineering, Universidade de Vigo, E-36310 Vigo, Spain; 2 UTINGAL S.L., Parque Empresarial Areas, E-36711 Tui, Spain

**Keywords:** epoxy resin, honeycomb panel, structural potting, automation, robotic milling

## Abstract

Structural potting is used to prepare honeycomb panels to fix metallic elements, typical in aircraft doors. In this paper, a full procedure for structural potting using robotic arms is presented for the first time. Automating this procedure requires the integration of, first, machining operations to remove the skin layers and prepare the potting points and, then, resin injection into the honeycomb cells. The paper describes the design, prototyping, and testing of specific end-effectors. Different end-effectors were explored to ensure efficient injection. The results obtained with the prototypes show that the potting quality is adequate to accomplish the required process checks for industrial manufacturing. The injection process time can be reduced by a factor greater than 3.5, together with the extra assets associated with the automation of complex tasks. Therefore, structural potting automation is demonstrated to be feasible with the end-effectors proposed for milling and injection, which are ready for use with conventional robotic arms in manufacturing lines.

## 1. Introduction

Parts made of carbon fiber-reinforced polymers (CFRPs) are a must in the present aeronautical industry, as a result of the need to limit energy consumption to reduce both the operational cost (essentially fuel consumption, directly related to structural weight) and the carbon footprint of the entire aircraft life-cycle. Design optimization studies try to make a systematic approach to overall aircraft design based on CFRP options [1]. This scenario obliges one to reconsider the whole cost chain in the manufacturing process of components, and studies have addressed large fuselage parts [2]. In recent years, the traditionally high-cost composite parts are have been revealed to be cost-effective in different industry fields [3,4], mostly driven by the reduction of the manufacturing cycle time by automating the processes, despite the costs of both the associated equipment and the raw material.

Using CFRP parts offers the opportunity and the need to reduce the number of joints in aircraft assemblies and design larger parts, with the best example being the case of the fuselage. This option, by rapidly increasing the footprint of the part, results in complex manufacturing operations, as well as requiring stock and assembly planning. There also exists a recurring problem in the aeronautical sector related to production times, and larger parts can help to reduce lead times. However, manufacturing CFRP parts is basically a manual, highly-skilled, and labour-intensive process, resulting in a high cost–rate balance. It is widely accepted that the only way to achieve the required production rates at limited costs is to automate some of the complex manufacturing processes for CFRP parts [5,6]. A well known case is that of automated tape laying machines. These are large and complex machines tailored to manufacture specific shapes; they are also very expensive and not capable of adapingt to the production of many different parts. However, the success of their deployment is based on both the productivity increment, which reduces the payback time, and in their making the manufacturing process time more stable, helping in production planning. Another sound reason for automation is the inherent repeatability of the process, making the failure rate more predictable and even lowering it drastically. Therefore, automation can directly reduce production costs.

The combination of automation of complex manufacturing and large parts brings specific challenges to production processes and requires complex planning, which has been presented by some authors as a ’process orchestration system’ [7]. These solutions usually include robotic arms, either fixed or mobile, to provide an extra-long stroke axis [8].

The suitability of using a heavy-duty robotic arm dedicated to machining CFRPs was studied for trimming [9] and milling [10], and quantified in terms of surface damage, delamination, and form deviations, depending on feed rate and cutting velocity. The general accuracy of robotic milling has been analysed by different authors [11,12]. Process planning including robotic arms is already integrated in software control packages for ISO 14649 and ISO 10303-238 [13].

The specific static and dynamic characteristics of robotic arms require dedicated planing of machining operations to improve accuracy by using a multi-axis compensation mechanism [14] and position control depending on force [15], kinematic analysis of the arm [16,17], or the effective stiffness of the arm [18]. Alternatively, a dedicated design of robotic arm for machining can improve the stiffness and dynamic properties, as well as the costs [19], and specific robotic designs [20,21] have been analysed to better understand their dynamic responses.

### Automating Structural Potting

Aircraft manufacturing includes some parts that require several production stages before assembly, which must be completed with very specialised procedures and become high-value parts. Aircraft doors and hatches are made with thick CFRP-honeycomb sandwich panels and require filling some regions with epoxy resins to make a monolithic point for fixing metallic elements, a process known as *structural potting*. The potting operation is typically carried out by hand, which requires trained personnel and creates a bottleneck in both the production chain and the productivity rate.

As explained in more detail in Section 2, structural potting basically consists of injecting epoxy resin into honeycomb panels, which is an activity that is very demanding in terms of manual procedures, is time consuming, and involves manipulating chemicals. Indeed, the resulting quality is highly dependent on the staff skills. Automating the potting processes would immediately reduce the process time, the wasted material, and the quality-default rate. At the same time, it would increase repeatability and manipulation safety. Therefore, automation would help in the overall process efficiency.

Automating this procedure requires the integration of, first, machining operations to remove the CFRP skin and prepare the potting points, and then, resin injection into the honeycomb cells. The procedure includes aggressive operations (removal of skin layers) and complex filling operations because the thick cores have different cell shapes, sizes, and depths. The structural quality depends critically on both the possible delamination and damage done to the skin layers and the proper integrity of the cells and solid filling of the cell channel with no voids, which would compromise the structural strength. Moreover, the structural quality and integrity of the whole part depends on the individual resin-filled points created, of which there can be many in the same part. Dealing with the complex steps involved in the potting process requires a skilled handcrafted procedure. Indeed, to ensure the quality of the potting, a post-process quality check based on some non-destructive (ND) inspection method is applied before accepting the part.

To the knowledge of the authors, there is only one commercial option offering solutions for robotic potting processes, from the company Airborne (Airborne Development BV, Den Haag, The Netherlands). Based on a robotic cell for resin dispensing, it is applied only into flat panels with reduced thickness [22,23]. The dispenser nozzle (ViscoTec Pumpen Dosiertechnik GmbH, Töging am Inn, Germany) controls the volume flow of the resin system, and was specifically developed to help the flow by adding hollow glass micro-spheres to reduce the density (Von Roll Holding AG, Breitenbach, Switzerland). This situation drastically reduces the range of possible applications, because the structural demands are limited due to the materials employed.

In this work, the automation of structural potting is demonstrated by using end-effectors developed for skin removal and resin injection. In Section 2, the study case selected in the paper is presented using a landing-gear door of the Airbus model A320. In Section 3, the different end-effectors are described. One prototype was developed for milling. Three designs are discussed for injection and two prototypes were tested for single-cell and vacuum-assisted multi-cell injection. In Section 4, the results of the tests conducted with the different tools are presented and discussed. For injection, both options were successful, with the process time being clearly favourable to the vacuum-assisted multi-cell injection option. The conclusions highlight that the end-effectors developed are a real full solution for the structural potting process automation.

## 2. Analysis Case: Potting of an Aircraft Door

Fuselage parts are a typical object of CFRP manufacturing, as shown in the early papers in the 1980s [24], where very few aircraft parts were considered for the new materials. Ref. [25] presented an Airbus A320 cargo door built with pre-pregs with the goal of reducing the manufacturing process cost. Large panels with curvatures adapted to the airplane’s envelope are typically manufactured as sandwich panels. The application of sandwich structures using fibre-reinforced polymers was already widespread in the 1990’s in many fields; however, the manufacturing techniques were badly developed and poorly documented [26]. Passenger and cargo doors, as well as hatches, include sandwich panels with a thick honeycomb core. However, the unavoidable drawback is the need for the insertion of fixing points for latches, hinges, guides, etc., which must be firmly attached to the panels. The skins are too thin to offer enough pull-out strength. The core, made of thin-walled cells, does not offer appropriate bulk material to firmly grab the inserted parts. The solution comes by locally converting the honeycomb into a monolithic part at the fixing points. This is achieved by filling the selected honeycomb volume with some structural resin material in an operation called ’structural potting’. The resin must be injected into the honeycomb so that the panel cell walls become the internal frame of the new structural monolithic volume. Note that the idea behind this is to create a solid insertion that is firmly connected to the whole panel and ready to be used as an anchoring volume to fix, e.g., metallic elements, including new drilling or threading operations in the new local potted region.

From the early work [27] about ’fully potted’ inserts in 1998, tests and numerical models (namely finite element models) have been used to study the pull-out strength of the metal insertions in thick CFRP laminates [28] or resin-filled cores in sandwich panels [29,30,31,32,33,34,35]. Based on the same type of analysis, some authors [36,37] developed parametric analysis for sizing the insert to the panel to help in the design stage.

**Figure 1:**
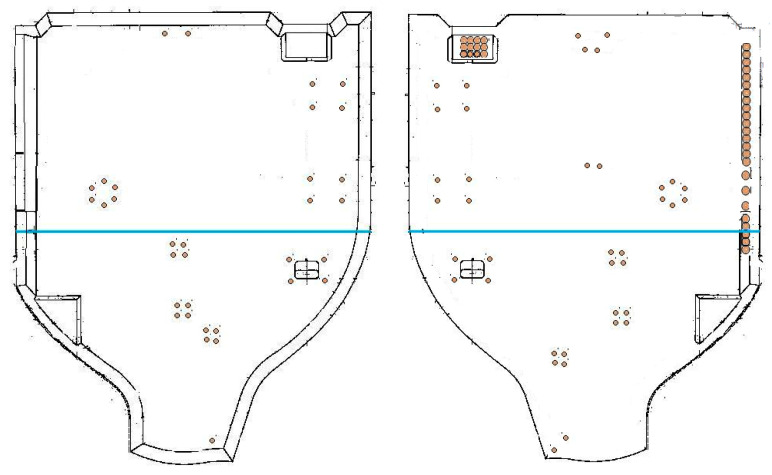
Drawing of the main landing-gear door of the Airbus A320 model: internal (**left**) and external (**right**) door faces. The overall size is about 1750 mm × 1680 mm. The positions of the 107 pocket milling operations (39 mm diameter) are marked as colour spots.

The case considered in this study corresponds to the structural potting of the main landing-gear door of the Airbus model A320. The door envelope is about 1750 mm × 1680 mm, 60 mm thick, and about 6 kg; see Figure 1. The honeycomb core is made of meta-aramid polymer impregnated with phenolic resin and cured (NOMEX ABS5676 G600, DuPont de Nemours Inc., Wilmington, DE, USA), with panel thickness 60 mm ± 0.25 mm and density 48 kg/m${}^{3}$. The door combines two types of cells with different sizes and shapes (so called types OX and EX HRH-10). The NOMEX honeycomb composite is a high-performance structural component of sandwich panels, preferred in applications for aircraft, aerospace, automobiles, etc. [38].

**Figure 2:**
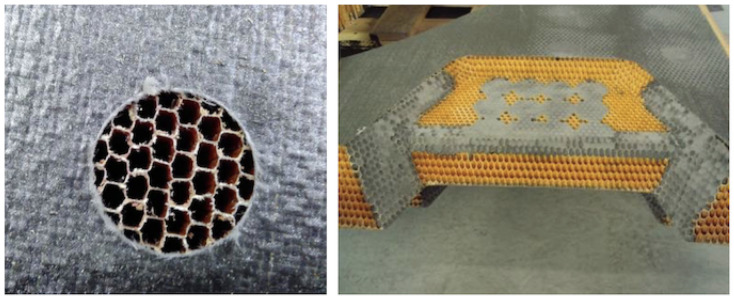
**LEFT**: Detail of the CFRP-skin removal by milling. The diameter is 39 mm. The cells of the core are visible and clean of burrs, ready for potting. **RIGHT**: Detail of the potting of one section corresponding to the upper part of the outer-side of the door. The core has been previously machined. The round spots as well as some sides of the honeycomb are then potted.

The sandwich part is subjected to multiple operations with the aim of locating the metallic parts on it. After machining, different spots of different shapes, depths, and sizes are potted to complete the hatch. Besides other actions, there are 107 pocket milling operations with 39 mm diameter each: 33 operations in the internal side and 74 in the external side, see Figure 1. The CFRP skin is removed by milling and the honeycomb cells are exposed; after removing the burrs (see Figure 2-left), it is ready for potting at the 107 locations, as well as in other regions. The spots will be potted by injecting epoxy resin in the cells, filling the honeycomb; see Figure 2-right. The procedure typically requires two operators and careful handling to ensure that the cell-walls are not damaged and voids are not created in the channels. Excess resin must be scraped and sanded down after curing.

The required structural quality requires using only accepted resins (according to the composite required qualification AIMS/IPS 11-01-005, EADS Airbus GmbH, Hamburg, Germany) and following an appropriate technical practice, including adequate curing procedures (according to the instructions for manufacture of monolithic parts with thermoset prepreg materials AIPI 03-02-019, EADS Airbus GmbH, Hamburg, Germany). Additionally, the CFRP skins are also checked to evaluate if any delamination appears just after milling, or if the honeycomb core cells have been damaged. All burrs must be removed before resin filling. The filling integrity can only be guaranteed by a dedicated inspection, as discussed in Section 4.

## 3. End-Effector Design and Prototyping

**Figure 3:**
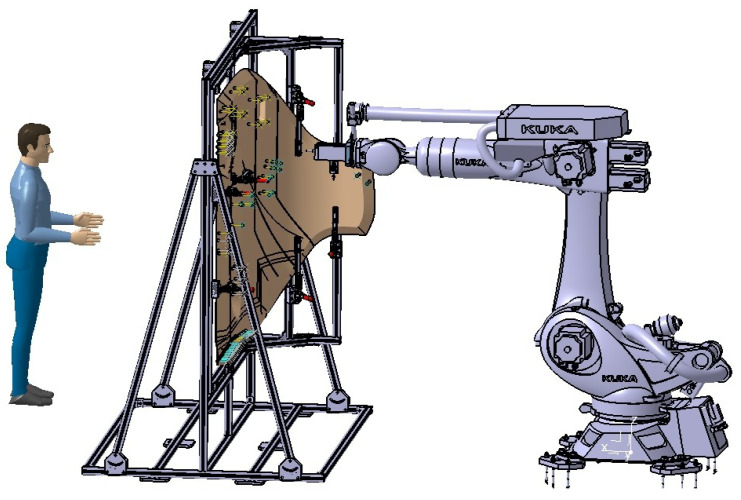
Drawing of the setup proposed to evaluate the case of machining and potting the 107 spots in the gear landing door by using specific end-effectors dedicated to each task, mounted on a conventional robotic arm.

To deal with realistic door structures with important thickness values involved (60 mm after curing the skin), curved envelopes, and many potting points (cf. Figure 1), the automation of the potting procedure requires solving two stages: skin removal and cell filling. The tools (end-effectors) developed for skin removal and resin injection in a processing line are presented in the next sub-sections. These end-effectors are ready to mount on typical robotic arms capable of reaching any spot of interest on the door; see Figure 3. The end-effector includes sensors to provide reference values to help fine-tune the operation. This capability offers the necessary feed-back information to complete the pre-defined arm trajectory adapted to the nominal part shape.

### 3.1. End-Effector Design for CFRP Skin Removal

**Figure 4:**
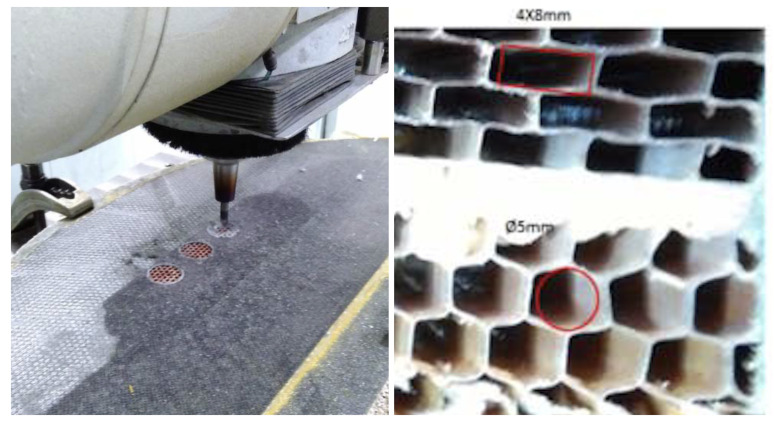
**LEFT**: Conventional milling operation to remove the CFRP skin of a sandwich panel made with the same cutting tool mounted in the designed end-effector. **RIGHT**: Detail of the honeycomb panel made of NOMEX, which combines two cell shapes and sizes under the skin.

The door under consideration has a CFRP skin made of one layer of polyester fabric peel-ply (100 g/m${}^{2}$) and one layer of biaxial (twill fabric) carbon pre-preg 12K (400 g/m${}^{2}$). The CFRP skin can be removed by a conventional milling operation in a separated process line; see Figure 4-left. The total skin thickness is about 0.6 mm. Removing the thin skin layers using a robotic arm requires fine control of the end-effector positioning. Besides the position calibration of the arm’s trajectories, a dedicated gauge to mark the surface level must be included in the end-effector for an effective process. The reason for this is that the nominal position in the projection plane is easy to achieve within a tolerance of 0.05 mm, provided by the trajectory control of the robotic arm. However, due to the curvy surface and manufacturing tolerances, the depth to remove the skin efficiently is not well defined, since the milling tool has to penetrate the surface normally to avoid delamination effects, as well as to avoid depleting the honeycomb cells.

**Figure 5:**
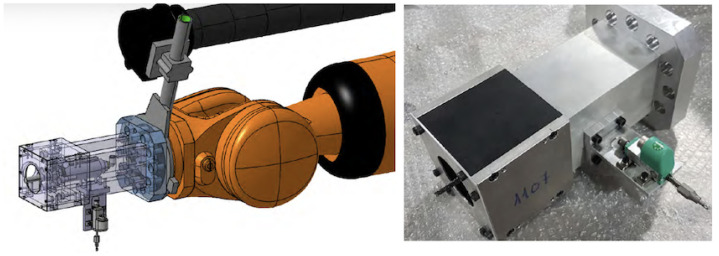
**LEFT**: Design of the end-effector for CFRP-skin removal. A rigid block connected to the robotic wrist holds a linear gauge and a spindle, located 90º from each other. **RIGHT**: Prototype of the end-effector.

The end-effector includes a compact spindle as well as a displacement gauge. It is located on the arm on top of the surface at the nominal position. The gauge (Potentiometric Displacement Sensor model 8712-10, Burster praezisionsmesstechnik gmbh & Co. kg, Gernsbach, Germany) touches and marks the surface reference to define the tool depth trajectory. The gauge provides a value with 0.01 mm resolution in typical ranges below 7 mm. The arm then relocates the milling tool according to the predefined path, using updated reference mark for depth. The design of the end-effector is based on a 90º alignment for the spindle and gauge. The spindle is set perpendicular to the robotic wrist link flange to provide maximum rigidity to the end-effector assembly while milling; see Figure 5-left. After locating the gauge and measuring the skin surface position, the end-effector is relocated with the milling tool facing the skin by a straight change of relative trajectory in the arm with the conventional control software.

This end-effector mounted on the robotic arm provides limited stiffness compared to a conventional milling centre. Furthermore, the milling parameters, mostly the torque–speed range of the tool, are much more limited. Indeed, the cutting tool must be a single-type choice to avoid tool changing operations. The end-effector must therefore be focused on the specific skin, hole size, and depth case considered. This is the situation in the case study, with 107 equal operations with a 39 mm diameter pocket milling. The cutting was carried out by circle interpolation to the specific diameter.

The CFRP skin removal was carried out using a solid carbide end milling tool with cutting diameter 10 mm and helix 25º (model JC870100, Seco Tools AB, Fagersta, Sweden).The tool holder was a shrink fit chuck HSK-A63 DIN 69893-1 (model A63.1409.10, Hoffmann Group, Munich, Germany). The electro-spindle (model ES951-A-0812-S, HSD SpA, Gradara, PU, Italy) had basic characteristics 22,000 rpm, 8 kW, 3.5 Nm, 13.9 A at 380 V.

The end-effector assembly design and prototype are shown in Figure 5. A single hollow cask, 230 × 145 × 145 mm${}^{3}$, made of aluminium 6061 T6, is directly fastened to the arm flange to provide a rigid frame to hold both the milling head and gauge, with a total weight of 27 kg. The end-effector requires the gauge power, signal, and pneumatic lines, and includes a vacuum nozzle to remove fibre debris, which are very damaging to all the equipment.

### 3.2. End-Effector Design for Single Cell Injection

The possibility of automating processes including epoxy filling or gluing requires developing specific resin systems that provide an effective solution, such as the recent solid system for gluing referred to in [39]. In this work, the same resin system used for manual processes was selected for the comparison study. Considering the need to fully fill the cells with resin, preventing any voids that would reduce the structural strength, the natural option is to consider direct injection into each individual cell. An end-effector dedicated to this task requires a thin long nozzle (straw-like) as an injector, which is first introduced inside each cell to a certain practical depth, which is then filled with resin while the injector is pulled out. Then, the injector moves to the next cell, repeating as necessary. It is also possible that panels will have cells with different sizes (Figure 4-right), requiring the replacement of nozzles adapted to each specific size, as well as changes in the injection-displacement velocity.

The potting is based on an epoxy resin dual component system. This requires mixing the two components in the right proportion, and mixing a homogeneous product ready to be used in the limited time while the viscosity is well suited for the injection stage. For the prototyping stage, the option was to develop an end-effector with a complete injector system. The end-effector includes a set of two cartridges for the epoxy components (model 7702971, 1:1 ratio, 2 × 300 mL, Nordson EFD, East Providence, RI, USA) and the mixing stage. The mixing is based on a static in-line double entry channel pipe. The mixing is based on the continuous flux of the resin components through the internal spiral elements. Therefore, the mixing quality depends on input control (for the right mixing proportions) and the pipe length (to ensure homogeneous conditions). To reduce the disposal of plastic consumables, a stainless-steel spiral tube mixer (model 7700126, Nordson EFD, East Providence, RI, USA) was selected. The use of cartridges requires mounting them and using an actuator to push out the product with pistons. The end-effector was equipped with a linear stage (model UA15-CNC, 300 mm stroke, SUHNER Schweiz AG, Bremgarten, Switzerland) and a servomotor (model 1FK7042-2AF71-1RB0, Siemens Aktiengesellschaft, Munich, Germany). The set can provide up to 3.5 cm${}^{3}$/s of resin at low speed (12 rpm), thus filling a hexagon cell sized as in Figure 2 and 60 mm long within 0.2 s. The actual rate is, however, imposed by the dispensing tip selected and the maximum dispensing pressure.

The tip must be long enough to go through the thickness of the cell. A compliant material would help to preserve the integrity of the thin wall cells during insertion. However, this kind of flexible material is not recommended for epoxy resins. Therefore, a precision stainless-steel tip 38 mm long with an internal diameter of 1.54 mm was selected (model 7018035, Nordson EFD, East Providence, RI, USA), demanding the correct alignment of the tip and cell to avoid any cell-wall damage. The injection rate limit is imposed by the resin viscosity and the maximum available injection pressure, resulting in a value of about 0.14 cm${}^{3}$/s and some 9 s per cell.

**Figure 6:**
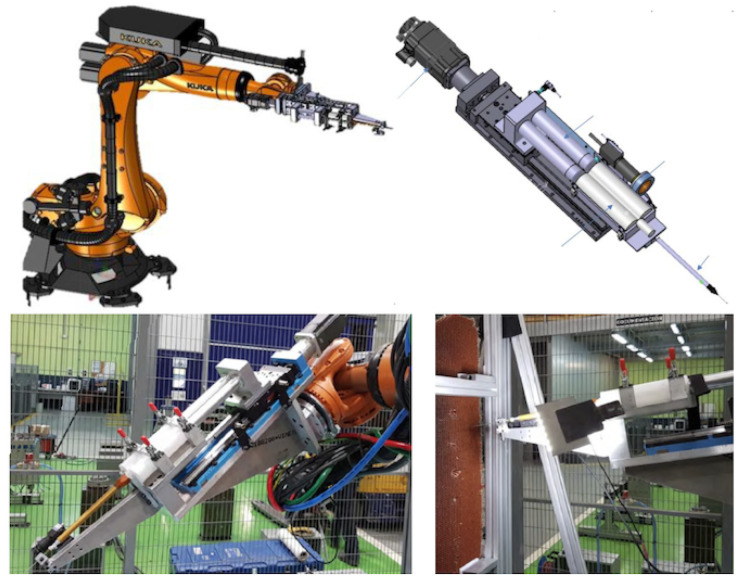
The prototype of the single-cell injector. **Upper panels**: Design of the injector group mounted on the linear actuator stage and end-effector mounted on the robotic arm. **Lower panels**: Injection group mounted on a rigid frame fastened to the robot wrist. The injection pipe is supported for rigid guiding. Test for filling the cells of a honeycomb panel.

The design of the end-effector thus includes one linear actuator, two cartridges, one mixing pipe, and the injection pipe. To help the resin flow, the natural solution is kept in an in-line layout, mounted with the long nozzle perpendicular to the flange of the robotic wrist. The resulting prototype is shown in Figure 6. The end-effector is mounted in a frame directly fastened to the arm flange. A plate supports the cartridges, the guide, and the actuator, weighting about 37 kg. For industrial deployment, a more effective solution would use a continuous supply of either the resin components or the mixed resin, requiring only the dispensing tip, thereby simplifying the end-effector and avoiding the replacement of any limited size cartridges.

The operation of this single-cell injector requires moving from one cell to another; this is fast because the movement is finished when the tip is just out of the cells. For the prototyping stage and tests, an additional optical camera (model CV-035M, Keyence Corp. America, Itasca, IL, USA) was used to monitor the process; see Figure 6. However, the critical parameter was to correctly tune the velocity to pull the pipe out of the cell, which is related to the resin injection speed. The goal is that the dispensed resin volume corresponds to the cell volume and no voids are created during the pipe extraction, along with maximizing the operation speed. The operation time for the single-cell injection is dictated by the time involved in the in-cell injection, which should be minimised while preserving the filling quality. The overall time is defined by the number of cells to be filled in; this is dictated by the injection time, with the cell-to-cell displacement being negligible. For these tests, the cells were filled in about 9 s and each 39 mm diameter spot with about 30 cells each required 4.5 min. Disregarding the transitions between spots, this is about 8 h per door with 107 spots to fill.

### 3.3. End-Effector Design for Multi-Cell Injection

The natural upgrade of the end-effector is a multi-cell injector. The time involved is directly reduced by the number of simultaneously filled cells. The nozzle now must be a multiple-tip nozzle, distributed in the honeycomb pattern and, ideally, compliant to help with insertion. Since the resin injection piston is unique, the only requirement is that the cells must all have equal internal volume, which is the case for the honeycomb panels in this study case. However, different regions may require a different nozzle; see Figure 4-right.

**Figure 7:**
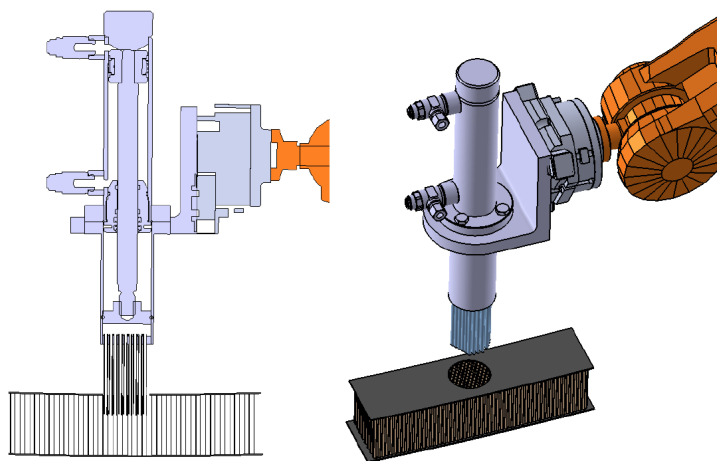
Design of the multi cell injection end-effector prototype. One single chamber for the resin, actuated by a piston, is filled with the mixed epoxy resin. The injector nozzle is made by a number of pipes fastened together in a circle. The pipe pattern must match that of the honeycomb below the skin at the potting spot.

The new injector with multiple tips requires increased resin volume ready for injection at an equal flow distribution. The mixing stage option as used for a single-cell is not suitable. For the multi-cell end-effector, it is better to use a single resin chamber driven by a piston to serve all the pipes at the same time, with the mixed resin prepared and filled previously in the chamber. Therefore, the end-effector only holds the injection stage using a more compact piston actuator. The end-effector uses a simple support to hold the piston set. The design is now with the piston axis set parallel to the arm flange; see Figure 7.

The multi-pipe injector nozzle was designed and virtually tested. The design is completely customised, based on a set of calibrated pipes bundled together to form the nozzle. The main drawback was that the cell pattern distribution under the skin may change from location to location, disturbing the insertion and compromising the integrity of the thin cell walls. Moreover, some cells could be not ’piped’ in the periphery because of the mismatch of the different cell patterns with the same nozzle; even worse, some empty cells could also occur in the internal circle, creating a faulty part. This virtual analysis showed that this option for multi-cell injection did not fulfil our needs and posed major drawbacks.

### 3.4. Vacuum-Assisted Multi-Cell Injection

The multi-cell injection concept is the key to creating an efficient approach to the cell filling process. Using multiple pipes, as mentioned above, would require the fine-tuning of the insertion process, adapting from position to position in the honeycomb core with the help, possibly, of image analysis. Alternatively, the resin can be directly injected at a simple surface spot, with the nozzle covering the whole pattern of cells. The injection end-effector is now largely simplified, reduced to only the resin chamber with the driver piston and the nozzle. Moreover, the position definition of the end-effector does not limit the process by using a (slightly) larger nozzle diameter than the skin hole. All the cells beneath the nozzle would be filled and the skin itself would define the nozzle injection border.

**Figure 8:**
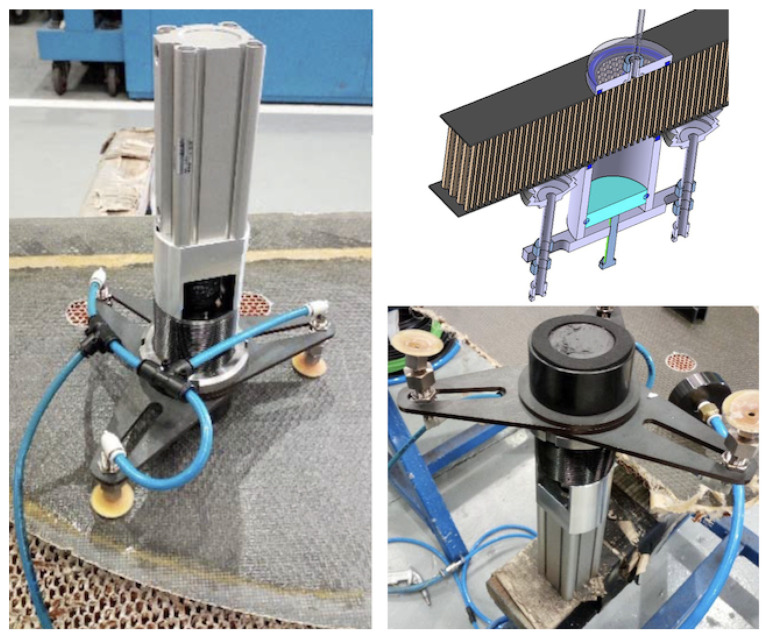
Vacuum-assisted multi-cell injector prototype. **LEFT**: The injection linear actuator and a resin chamber are located on top of the panel with three vacuum grips on an ad-hoc frame. **RIGHT**: The chamber-nozzle is a hollow cylinder filled with resin. The nozzle interface is a flat foam ring, mounted as a gasket. The vacuum nozzle used to assist the filling is located on the opposite side of the panel.

The injector prototype was mounted and tested by using a linear actuator (model CDQ2B32TF-75DZ magnetic piston, 15 bar, SMC, Chiyoda City, Japan) to actuate the piston of the resin chamber (with a rod and plunger system made of hardened steel (DIN St 37-2)). The chamber-nozzle set was simply a hollow cylinder filled (manually) with resin. The actuator-chamber set was mounted with an ad-hoc frame, using three vacuum grips to hold it firmly to the skin surface while injecting the resin; see Figure 8. The nozzle included a simple interface made of a flat foam ring mounted as a gasket to avoid the resin spilling out around the head.

The drawback of this method is that the cell filling now depends only on the forced flow from one side. The nozzle remains static and does not help in the fluid distribution, opposite to the effect of using pipes for the injection. The overall effect is that voids can be more easily created, caused by the resin viscosity and the ’sticky’ effect at the cell walls. To help improve the resin distribution, a vacuum nozzle is paired at the opposite side of the honeycomb panel. The resin is thus under the double effect of the injection pressure from one side and under-pressure from the opposite side of each cell channel. Voids are rarely formed if the injection flux is not too fast. The nozzle gasket is also key to guaranteeing that the skin–nozzle interface makes a tight volume so that the vacuum is applied effectively to fill the cell group. This injection option, tested with the prototype shown in Figure 8, resulted in no empty cells observed in the many trials conducted in the workshop once the injection parameters were tuned correctly.

**Figure 9:**
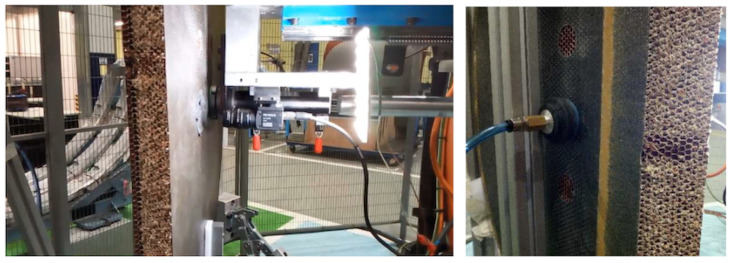
Test of the vacuum-assisted multi cell injection system. **Left**: The end-effector is located at one side of the panel by the robot arm. The optical camera and light-panel help the operation. **Right**: A vacuum nozzle is located at the opposite side of the panel to assist the resin injection.

The vacuum-assisted multi cell injection system was then mounted on a robotic arm and tested on a real setup; see Figure 9. The injector was mounted with the same linear stage system as with the single-cell injector, and with a camera vision system to actuate the piston. The procedure requires acting on both sides simultaneously, injecting the resin from one side and assisting with vacuum on the opposite side. This procedure could require removing the laminate skin on both sides, if present, and using robotic arms on both sides. In this study, the potted spots had CFRP skin on only one side, with the core open on the opposite side, thus creating a convenient situation. The end-effector was mounted perpendicular to the flange of the robotic wrist. A continuous resin supply was included to re-fill the chamber; see details in the Appendix A. During the tests, the end-effector also included an optical camera. The image was used for control: first, to correctly position the nozzle on top of the skin opening and then, prior to the procedure, to check the cleanliness (burrs) of the honeycomb cells. Finally, after filling, the image served as a post-check. The time involved in the tests made was about 1.3 min per spot; this is 3.55 times faster than single-cell injection.

## 4. Experimental Results

In order to verify the correct functioning and compare the performance of the prototypes, several tests were conducted, repeating and adjusting the process as many times as necessary to obtain the right test results to use as references for the comparisons. The prototypes of the end-effectors developed for the automation were mounted on the robotic arm and the panels were subjected to milling and injection operations to test the process and compare the options. On the one side, the CFRP-skin removal was tested to verify the integrity of the sandwich panel. On the other side, the resin filling result was compared using the different options developed.

### 4.1. CFRP-Skin Removal

The process for machining CFRPs is well documented and is a common process in the aeronautical industry. This case refers to the removal of a thin skin (0.6 mm thick, two layers) glued to the honeycomb panel. The process is carried out by a conventional milling machine (see Figure 4-left) with a high rate of success. The milling of CFRPs is known to relate the cutting method and tools with the forces and the surface quality [40,41,42]. Different strategies and methods have been studied to improve the milling performance, typically in respect to the delamination issue [43,44]. Model analysis has also been used to include tool wear and milling forces in order to better master the process parameters [45,46,47]. The milling process parameters can also be specifically defined for a robotic arm implementation. In [48], the authors found that spindle speed dictates the surface roughness, while milling depth dictates the vibration. The problem of milling parts with low rigidity is a common topic in the aircraft industry, requiring vibration-controlled approaches [49].

The main concern with this operation is the potential damage caused to the layers, namely due to delamination. It is also possible to damage the honeycomb core if the cells walls are pulled by the cutting tool and the cell-net continuity is broken. Furthermore, the burrs, if any exist, must be removed. The specifics of cutting honeycomb panels are fine-tuned using the results on tearing, rubbing, and cell-wall integrity [50], and specific solutions using cooled machining have been recently proposed [51]. A proper milling operation that just removes the CFRP-skin, with only a tiny fraction of honeycomb cells removed, avoids burr formation (to the maximum (tiny) allowed size), as well as any cell degradation. This is possible if the mill depth is carefully fit to the skin thickness, as the new end-effector allows with the position gauge.

**Figure 10:**
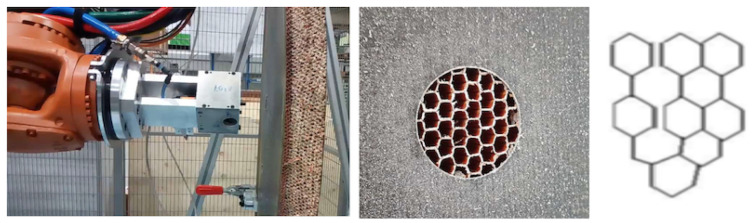
**LEFT**: Picture of the end-effector prototype dedicated to the CFRP-skin removal operation mounted on the robotic arm and ready for the milling. **MIDDLE**: The result of the skin-removal for 39 mm diameter milling. The core cells are exposed, preserving the cell integrity, and without burrs, ready for potting. **RIGHT**: The integrity of the core can be compromised if cell walls are split or damaged, as in the drawing. This can be the result of the insertion of the resin injectors or due to milling with blunt tools or too deep into the core.

The result of the skin removal operation is shown in Figure 10. The cutting was conducted by circle interpolation and it took about 27.5 min to complete the skin removal of the door, with some 15.4 s per spot (39 mm). The post-operation check was completed by visual inspection. All tests conducted with the end-effector showed that the milling result is perfectly adequate to the quality requirements. No delamination was observed in the many tests carried out with the end-effector, using sharpened tools and the selected spindle. The measurements taken with the gauge allowed appreciable burrs to be avoided and the cell integrity to be preserved. In a processing line, an optical camera would allow for the control of both skin delamination and core (burrs and integrity). Alternatively, image control can be utilized in the next step, prior to potting, when cameras are used to verify the injection stage.

The time involved in the milling operation is comparable between the end-effector mounted on the robotic-arm and a conventional milling machine. The main difference is that working in a process line avoids the logistic chain (stockpiling and delivering) due to a separated process. This is an important part of the time and cost savings, and is made possible with the automation solution.

### 4.2. Resin Injection: X-ray Radiographic Non-Destructive Inspection

The quality of the filling for an acceptance test is defined not by load tests to probe, e.g., the fixation pull-out strength, but by practical manufacturing aspects, namely, verification of the characteristics of the resin used, the curing process control, and the correct filling of the cells. Therefore, quality assessment places importance on the proper manufacturing procedures, with the appropriate functionality justified by independent structural analysis and tests; see Section 2. Concerning the evaluation for this study, the critical check is correct cell filling, since the resin and curing are those specified by the client and manufacturer, respectively, according to the specific regulations for the aeronautic industry.

**Figure 11:**
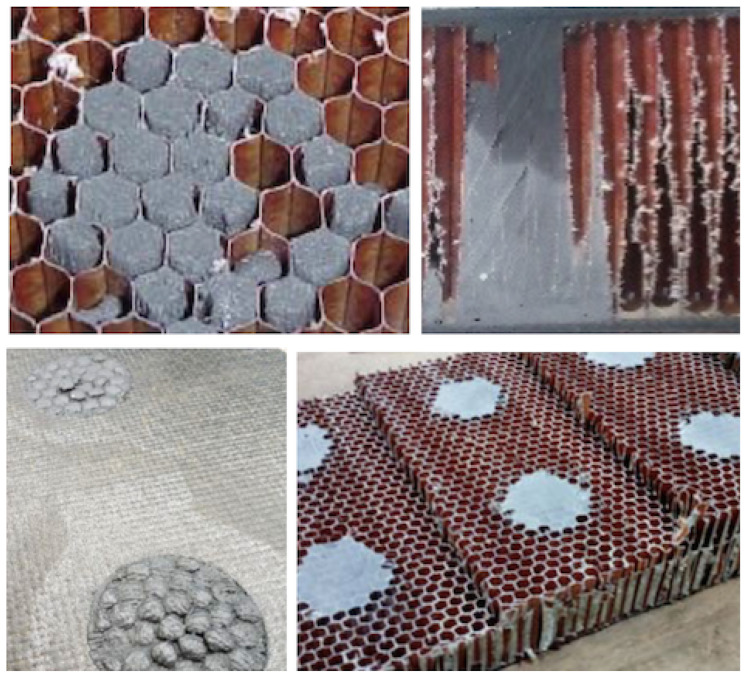
**UPPER-LEFT**: The potting results are faulty if one or more cells are empty in the central part, or if the cells are not completely filled. Both cases are clearly visible in this picture. **UPPER-RIGHT**: The cutting of the panel allows for the direct inspection of partially empty internal cells, as in the picture. **LOWER-LEFT**: The resin must overflow the cells to ensure their proper filling. **LOWER-RIGHT**: The potted spots are grounded to complete the potting.

The potted spots are typically positions to drill fixation points for some kind of fastener. Therefore, the structural integrity of such fixing points requires that there are no empty cells close to the central drilling bore, nor cracks or voids in that volume. In Figure 11 upper-left panel, a faulty potting spot shows empty cells in the central part, as well as partially filled cells, with void space around the walls. The resin must overflow to avoid such partial cell filling (Figure 11 lower-left panel). An optical camera picture can be used for this kind of checking. However, this visual inspection does not help to control the internal volume. In Figure 11 upper-right panel, some voids are visible in the inner volume, as observed after cutting the panel. This kind of test can help to tune the manufacturing (injection) parameters.

The panels must be examined and qualified individually; thus, ND methods must be applied to preserve the integrity of the whole part. Different ND methods are available and capable of spotting different issues on CFRP parts for aircraft [52,53,54]. X-ray radiographic methods are a well-proven ND tool. It is possible to obtain 2D (projections) and 3D images (tomography) based on the radiation absorption properties of the different materials, and thus analyse CFRP parts to different detail levels [55,56]. Indeed, these methods are proven valid for adequately controlling volumetric defects [57].

The brightness scale observed in a radiographic 2D image is related to the radio-density of the materials involved, as well as their thicknesses. In this case, it is closely related to the thickness of the material traversed by the X-ray beam, since all the involved materials (skin, honeycomb, and filling resin) are, mostly, carbon-based, with close radio-densities. As the cell-wall and CFRP-skin 2D projection are very homogeneous, the brightness level (or grey scale level) is a direct index of the total traversed matter. Therefore, if voids are present in the cells, the air amount will change the grayscale value on the projected image, due to the different (radio-)density with respect to a fully filled cell.

**Figure 12:**
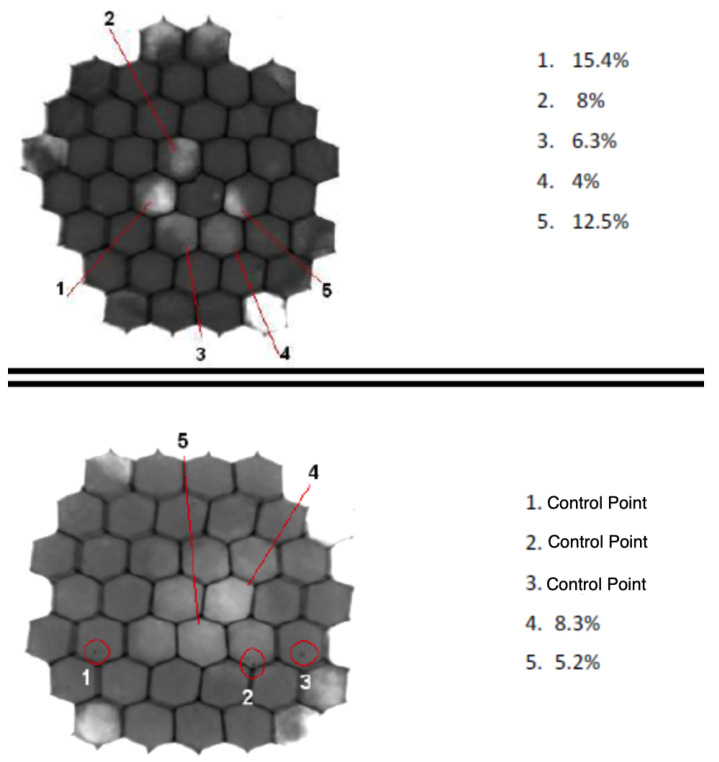
X-ray analysis of two potted spots, each corresponding to different panel thicknesses. In each case, cells with spots showing brightness deviations are marked.

To test the potting filling quality, several samples were prepared with the vacuum-assisted injection method and analysed with X-rays. To finish the potting process, the spots must be grounded (Figure 11 lower-right panel). This is also important to homogenise the X-ray analysis, which directly depends on the panel thickness value. The samples were analysed with a 160 kV X-ray tube and the images were recorded with a flat panel of 1024 × 1024 pixels, with pixel size 1 × 1 μm${}^{2}$. In Figure 12, the results of two different faulty potting spots are shown. They correspond to two different core thicknesses; thus, the grayscale differs for each case. However, both were calibrated with respect to the corresponding fully-filled cell reference case. The grayscale quantitative analysis allows for defining the deviations in brightness value. Some cells show deviations in the range from 4 to 15.4%. The deviations can affect the region of the cell (cases 1, 2, 3, 5 in the upper panel case) or the overall projection of the cell (case 4). An independent test with reference samples allows one to set a numerical limit on the accepted deviation, correlated with the actual size of the void, depending on the ratio of the radio-density of the resin and air and on the void size. In the lower panel, the density deviations are marked (cases 4 and 5), as well the presence of spots (cases 1, 2, 3) generated for the size calibration, similar to the image quality indicators ISO 19232-1:2013 based on metal wires, which serve as size reference gauges.

The multiple tests developed in this ND program demonstrate that it is feasible to achieve a repeatable fully successful injection procedure by adjusting the injection flow rate. For single-cell injection, the final quality depends on carefully tuning the injection flow rate and pipe extraction speed. For multiple-cell injection, it depends mostly on the bubble-free quality of the resin (see Appendix A), because the injection itself is continued until overflow. As seen from the measurements, voids are rarely left in the internal cell volume if the injection speed is kept steady and adapted to the viscosity of the resin (which dictates the maximum speed of the process), and the resin mixing is bubble-free.

Since the vacuum assisted multiple-cell injection proved capable of making the correct cell filling and is up to 3.55 times faster, it is the selected automation solution to implement structural potting. The success rate observed is promising, and requires a dedicated specific sampling analysis to define the qualifying procedure. Indeed, (almost) the only possible source of issues would be a failure in the resin supply system due to non-steady flux or viscosity of the resin, or to bubble content.

## 5. Conclusions

The demanded increase in production rate at a limited cost across the aeronautical industry requires the automation of the manufacturing processes related to large CFRP parts. In this work, we developed prototypes of tools capable of automating one complex task: the structural potting of honeycomb panels. The specific end-effectors designed and tested here allow for, first, removing the CFRP skin of the panels and, then, filling the panel cells with resin to create a monolithic volume where necessary. The designed end-effectors are ready to be mounted on conventional robotic arms in a production line, as tested.

- The end-effector dedicated to the CFRP skin removal proved capable of proper operation, with the rigidity of the robotic arm high enough to guaranty the milling quality. The tests performed showed no CFRP delamination defects and the core (cells) integrity was preserved.
- The resin injection operation required considering different options to fill either a single cell or multiple cells. Specific prototypes were tested. X-ray radiographic inspections performed on the samples showed that it is possible to obtain the right filling quality with the two types of end-effectors after properly tuning the injection rate. The vacuum-assisted multi-cell injection end-effector, with a simplified design, provided the correct filling quality and reduced the operation time by a factor of more than 3.5.

The deployment of this end-effector system for potting in a processing line requires a first stage with one robotic arm to remove the CFRP-skin on each panel side affected by this operation. The second stage includes a robotic arm with the injection end-effector. The paired vacuum nozzle at the opposite panel side requires a second arm. An alternative option would be using co-bots for this position. The operator would assist the operation and, at the same time, conduct the inspection control of the correct resin dispensing cycle. The possibility of automating the structural potting of large aeronautic (thick) honeycomb panels is proven feasible with this innovative end-effector system.

## Figures and Tables

**Figure A1:**
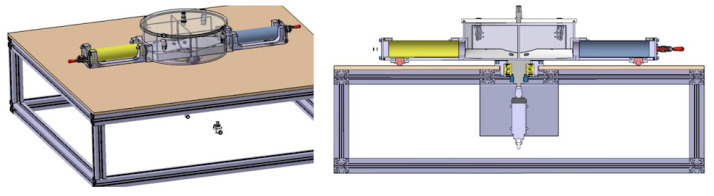
Drawing of the bench dedicated to mixing the bi-component resin system. The chamber is evacuated, causing the cartridge to discharge into the chamber. The paddle mixer and continuous vacuum guarantee correct, bubble-free mixing.

## Data Availability

Not applicable.

## Appendix A. Resin Cartridge Filling

The mixing of the bi-component resin system is necessary before injection. For the multi-cell injection option, the mixing must be performed separately so that enough resin volume can be provided to re-fill the resin chamber prior to injection. The mixing requires (i) the right component combination, (ii) the complete mixing of the components, and (iii) elimination of any bubbles in the mixed product.

A dedicated mixing bench was set up to provide the resin; see Figure A1. A chamber with two opposite inlets admits each resin component, supplied in individual cartridges, which can be adapted to any commercial size. The closed chamber is evacuated, therefore emptying the cartridges. The resin system is for mixing equal volumes, simplifying the operation. A simple paddle mixer, actuated by an external engine, shakes and mixes the resin. The continuous vacuum easily removes the bubbles from the resin volume. A different outlet is used to pump the mixed resin into the end-effector nozzle.
